# Assessing the association between health promotion initiatives and student nutritional status and lifestyle: a regional observational study from Italy

**DOI:** 10.3389/fpubh.2026.1750624

**Published:** 2026-02-16

**Authors:** Claudia Veronica Carletti, Eleonora Maurel, Federica Concina, Manuela Giangreco, Alessandra Knowles, Luca Ronfani, Paola Pani, On behalf of the OKkio alla Salute FVG group, HBSC FVG group

**Affiliations:** Clinical Epidemiology and Public Health Research Unit, Institute for Maternal and Child Health-IRCCS Burlo Garofolo, Trieste, Italy

**Keywords:** cross-sectional study, HBSC, OKkio alla Salute, physical activity, school health promotion practices, school policies, sedentary behaviour

## Abstract

**Background:**

Schools are widely recognized as key settings for health promotion interventions to address excessive weight among children and adolescents. This cross-sectional observational study evaluated the association between the presence or absence of health promotion practices in a representative sample of primary and secondary school classes in the Friuli-Venezia Giulia (FVG) region and students’ weight status and dietary and physical activity (PA) habits.

**Methods:**

Data on nutritional status, dietary and PA habits, and on school health promotion initiatives, with or without family or local health unit (LHU) involvement, were derived from questionnaires completed by school principals, students and families as part of the 2023 OKkio alla Salute (children aged 8–9) and the 2022 Health Behaviour in School-aged Children (adolescents aged 11–17) surveys.

**Results:**

The prevalence of overweight or obesity was 25.1% among primary school children and 16.7% among adolescents. Most primary schools implemented nutrition (86.5%) and PA (97%) education, but with limited family and LHU involvement. The only significant difference was observed in eating habits, with higher consumption of adequate mid-morning snacks in children attending primary schools that implemented healthy eating initiatives involving parents (*p* < 0.001). In secondary schools, most of which reported implementing policies on nutrition (92.8%) and PA (97.3%), the only significant difference was fruit consumption ≥ once/day in adolescents attending schools with nutrition-related policies.

**Conclusion:**

While school-based health promotion practices and policies are widely implemented in the FVG region, their association with students’ nutritional status, dietary habits and PA was limited. These findings highlight the limited benefits yielded by existing school practices and policies and underline the potential importance of stronger parental involvement, multi-sectoral collaboration and more structured school health policies.

## 1 Introduction

In Europe, overweight and obesity in childhood and adolescence remain major public health challenges. According to the World Health Organization (WHO) European Childhood Obesity Surveillance Initiative (COSI), about one in three children aged 7–9 years is overweight or obese in several countries across the WHO region (1). Italy, in particular, reports some of the highest rates in Western Europe: data from the Italian surveillance system “OKkio alla Salute” indicate that in 2023, 29.9% of children aged 8–9 were classified as overweight or obese (20.1% overweight and 9.8% with obesity), with higher prevalences in the southern regions and among children from lower socioeconomic backgrounds (2). A similar pattern can be observed during adolescence. Data from the 2022 Health Behaviour in School-aged Children (HBSC) survey indicate that more than one in five adolescents in the WHO European Region is affected by overweight or obesity (3). The prevalence is higher among boys (27%) than among girls (17%) and disproportionately affects adolescents from less affluent families (27%) compared to those from wealthier backgrounds (18%). In Italy, 22.6% of adolescents are classified as overweight or obese (18.2% overweight and 4.4% obese), with higher rates consistently observed among males across all age groups and with a decreasing trend as the age increases.

One key recommendation of the WHO report on Ending Childhood Obesity (4), draws upon data from previous intervention studies to highlight the role of schools in reducing youth obesity, by implementing comprehensive programs that foster healthy school environments and enhance health and nutrition literacy and physical activity (PA) among school-age children. These programs should be characterized by a wide-ranging scope, cost-effective policies, a social perspective, and a multi-sectoral and multi-level approach (5, 6).

Schools where children and adolescent are exposed to a supportive environment, i.e., with health policies, PA and nutrition education, and physical education (PE) classes during school hours (7), offer an ideal environment for influencing health-related behaviours at a time in which habits are still being formed (8, 9). They are where students spend a substantial portion of their lives and therefore interventions can attain high coverage quickly (6), and where socioeconomic inequalities generally carry less weight than in other social environments, such as the community or sports clubs (10).

In addition to nutrition education classes and regular PE classes, there are other factors in the school environment that can influence healthy behaviours: the quality of available food and beverages, including lunch which, in several countries, is provided by the school, the implementation of extracurricular sports programs and initiatives such as safe walking and cycling routes and walking school buses (11).

Several key factors have been identified in the literature as capable of influencing school-based interventions designed to prevent obesity in children and adolescents. Recent studies have shown that socioeconomic and cultural factors, school policies and the physical environment (6), in addition to teacher and staff engagement and the involvement of parents and caregivers in the interventions, can determine the success of such interventions (12–14).

This cross-sectional study explores the associations between the presence or absence of school-level health promotion practices and policies, as reported by school principals, and students’ nutritional status and lifestyle behaviours using FVG data from two National Surveillance Systems on childhood and adolescence.

## 2 Materials and methods

This cross-sectional observational study is based on surveillance data derived from two surveys conducted in FVG: the 2023 wave of the OKkio alla Salute survey and the 2022 wave of the HBSC study.

### 2.1 OKkio alla Salute survey

OKkio alla Salute is an ongoing, population-based survey, conducted every 4 years, that monitors the health and health-related behaviours of primary school children across all 21 Italian regions. It is coordinated by the Istituto Superiore di Sanità - Italian National Institute of Health (ISS), with the collaboration of regional health authorities and the Ministry of Education and Merit. The target population consists of third-grade primary school children, typically aged 8 to 9 years. Italy also contributes these data to the WHO European Region’s COSI (1), enabling international comparisons on children’s health behaviours and nutritional status.

The sampling strategy follows a stratified cluster design, as recommended by the WHO (15), using school classes as the primary sampling unit. Sample selection is carried out at the Local Health Units (LHUs) level, based on lists of third-grade classes of all public and public-private primary schools provided by regional school authorities. For each school, the probability of having their classes extracted is proportional to the number of third-graders enrolled. All children in selected classes are invited to participate. Regions may choose to design samples that are representative of either the entire region or individual LHUs. Sample size calculations are based on previous estimates of excess weight prevalence, aiming for a precision of 3% at regional level and 5% at LHU level. These estimates are further refined by adjusting for design effects observed in previous survey waves.

Data collection involves three sets of questionnaires, completed by children, their parents or caregivers, and school principals (Supplementary Table S1), alongside direct anthropometric measurements of weight and height taken by trained healthcare staff. The detailed survey protocols and data collection procedures are described elsewhere (16, 17).

### 2.2 HBSC survey

The HBSC survey is an international, multicentre study conducted every 4 years in collaboration with the WHO Regional Office for Europe. In the 2021/2022 round, the survey was implemented in 44 countries and regions across Europe, Central Asia, and Canada. In Italy, the 2022 edition was coordinated by the ISS and the Universities of Turin, Siena, and Padua, with support from both the Ministry of Health and the Ministry of Education and Merit. The HBSC targets adolescents aged 11, 13, 15, and 17 years, capturing data on self-reported anthropometric measures (weight and height), lifestyle behaviours, health outcomes, and social and school environments (3).

The sampling follows international HBSC guidelines and employs a systematic cluster sampling method. School classes serve as the primary sampling unit, and the selection is based on national lists of schools provided by the Ministry of Education and Merit. As with OKkio alla Salute, students and school principals complete standardized questionnaires (Supplementary Table S1). Additional details regarding the sampling methodology and data collection process are available in Inchley et al. (18).

Both surveillance systems (OKkio alla Salute and HBSC) operate under strict ethical standards. The study protocols for OKkio alla Salute and HBSC were reviewed and approved by the Institutional Ethical Board of the ISS.

### 2.3 Data

The present study examines how a school environment that self-assesses as supportive is associated with selected lifestyle indicators among children and adolescents.

School environment information was derived from the school principals’ questionnaires in both surveys. In the case of OKkio alla Salute, we evaluated the presence of school health promotion practices related to healthy eating and PA, including curricular programs and projects involving parents or LHUs. The HBSC survey assessed whether schools adopted health promotion policies or guidelines, and whether curricular activities on nutrition and PA were present. Information on timing, duration, intensity, implementation fidelity and content of the reported practices was not collected.

Lifestyle data included indicators related to weight status, dietary habits, PA, and sedentary behaviours. Body Mass Index (BMI) was calculated for each participant using measured (OKkio alla Salute) or self-reported (HBSC) height and weight, and categorised according to the age and sex-specific international thresholds developed by the International Obesity Task Force (IOTF) (19, 20). Based on these thresholds, children and adolescents were classified as underweight, normal weight, overweight, or obese.

Dietary habits among younger children (OKkio alla Salute) were assessed through questions on consumption of: (1) breakfast, classified as no, yes/inadequate, yes/adequate; (2) mid-morning snack, classified as no, yes/inadequate, yes/adequate; (3) fruits and/or vegetables ≥5 times a day (yes/no); (4) sugary and/or carbonated beverages every day (yes/no). Breakfast and mid-morning snack quality were determined using criteria established by the Centre for Research on Food and Nutrition (CREA) (21), with “adequate” breakfast defined as one containing proteins, complex carbohydrates, and simple carbohydrates. Mid-morning snacks were considered adequate if they included healthy items such as fruit, yogurt, or unsweetened juice and provided around 100 calories. Children were asked to recall what they had consumed that morning to determine dietary quality, following the framework established by Ciardullo et al. (22).

In the HBSC survey, dietary habits were captured via adolescent self-reports. Relevant indicators included: (1) breakfast consumption during the school week (yes/no); (2) fruits consumption (≥ once a day); (3) vegetables consumption (≥ once a day); (4) daily consumption of sugary and/or carbonated beverages (yes/no).

PA habits were also analysed using age-appropriate indicators. In OKkio alla Salute, children reported whether they had been physically active (sports, outdoor play or PE at school) in the last 24 h, and parents reported whether their child had engaged in outdoor play or sports the day before the survey and if the child habitually walked or cycled to school. HBSC assessed adolescents’ PA levels through questions on moderate and vigorous exercise, based on WHO recommendations (23). Adolescents were asked about the frequency of (1) moderate PA for at least 1 h, on a scale of 1 to 7 days/week, and (2) vigorous PA (substantial increase of heart and breathing rates), on a scale of 1 to 7 days/week.

Sedentary behaviours were captured in both surveys through questions on screen time. Parents reported how much time their child typically spent watching TV, playing video games, or using digital devices on a school day. This information was categorized into two groups: ≤2 h or >2 h, consistent with WHO recommendations (23). The questionnaire also included a question on the presence of a TV in the child’s bedroom, as this has been linked to reduced parental oversight (24).

Socioeconomic status (SES) was also considered. In the OKkio alla Salute survey, the mother’s education level served as a proxy for family SES, a measure commonly used in public health research for its strong association with children’s health outcomes (25). In the HBSC survey, SES was measured using the Family Affluence Scale (FAS), which captures material wealth and family lifestyle. The FAS includes items such as car ownership, number of computers, availability of a dishwasher, private bedroom for the adolescent, number of bathrooms, and frequency of family vacations abroad (26).

Due to the differences in sampling strategies, measurement methods (measured vs. self-reported anthropometrics), age groups, and years of data collection, the data on children and on adolescents are not directly comparable. Therefore, results are interpreted within each surveillance framework rather than comparatively, and conclusions focus on patterns observed within each dataset.

### 2.4 Statistical analysis

Descriptive analysis used frequency and percentage for categorical variables, median and interquartile range for continuous ones. The Chi-square or the exact Fisher test was performed to evaluate associations between two categorical variables, as appropriate. *p*-value < 0.05 was considered statistically significant. SAS software 9.4 (SAS Institute Inc., Cary, NC, USA) was used for statistical analysis.

## 3 Results

In the OKkio alla Salute regional survey 2023, 79 classes were sampled, 7 of which in public-private schools, with a mean size of 18 children for each class, for a total of 1,376 children aged 8–9 years. Data were analysed for the 1,174 children who were in school on the day of the interview and whose parents gave consent to participate and completed the questionnaire. The questionnaire was predominantly completed by the child’s mother (83%), and less frequently by the father (16%) or another caregiver (1%). Overall, as reported in Table 1, 57.8% of children were 8 years old and 51.4% were male. Most children (69.7%) lived with both parents, 39.4% had mothers with a high level of education, and 41.8% had mothers who worked full time.

**Table 1:** Sample characteristics from the OKkio alla Salute and HBSC surveys.

Characteristics	OKkio alla Salute		HBSC	
Age, *n* (%)				
	≤7 y/o	4 (0.3)	11 y/o	1,021 (26.2)
	8 y/o	668 (57.8)	13 y/o	1,047 (26.9)
	9 y/o	475 (41.1)	15 y/o	1,052 (27.0)
	≥10 y/o	9 (0.8)	17 y/o	772 (19.8)
	Total	1,156	Total	3,892
Sex, *n* (%)				
Male		601 (51.9)		1891 (48.6)
Female		556 (48.1)		2001 (51.4)
Total		1,157		3,892
Mother’s educational level, *n* (%)				
Low		201 (17.2)		—
Medium		509 (43.5)		—
High		461 (39.4)		—
Total		1,171		—
Mother’s employment status, *n* (%)				
Full-time		415 (41.8)		—
Part-time		357 (35.9)		—
Not employed		222 (22.3)		—
Total		994		—
Living situation, *n* (%)				
Mother and father		841 (69.7)		2,368 (81.0)
Single parent		116 (9.6)		361 (12.3)
Blended families		16 (1.3)		71 (2.4)
Other		234 (19.4)		125 (4.3)
Total		1,207		2,925
FAS, %				
Low		—		18
Medium		—		53.3
High		—		28.7

FVG region, 2022–2023. *n*, number of subjects; %, percentage; FAS, family affluence scale.

In the 2022 HBSC survey, of the 269 classes sampled, 263 accepted to participate to the data collection, 6 of which in public-private schools, with a mean size of 20 adolescents for each class. The 3,892 adolescents who completed the questionnaire with the parents’ consent, were equally distributed by age (20%–26% in each age category) and sex (51.4% female), and in most cases (81%) lived with both parents (Table 1).

Table 2 shows the distribution of BMI categories and levels of PA among the OKkio alla Salute and HBSC survey participants. In OKkio alla Salute, 25.1% of children had excessive weight (18.2% overweight and 6.9% obese), without significant differences between males and females (*p* = 0.59).

**Table 2:** Prevalence (*n*, %) of BMI categories and PA levels among children (OKkio, 8–9 years) and adolescents (HBSC, 11–17 years) in the Friuli Venezia Giulia region, 2022–2023, overall and stratified by sex (and by age group for HBSC).

Characteristics	OKKIO alla Salute Age 8–9 years *n* (%)					HBSC Age 11–17 years *n* (%)							
	Sex					Age				Sex			
	Total	Male	Female	Effect-size	*p*-value	11	13	15	17	Male	Female	Effect size	*p*-value
No. of children, *n* (%)	1,157	601 (51.9)	556 (48.1)	—	—	933 (25.0)	980 (26.3)	960 (25.7)	860 (23.0)	1836 (49.2)	1897 (50.8)		—
BMI categories, *n* (%)													
Underweight	19 (1.7)	10 (1.7)	9 (1.6)	0.04	0.59	37 (4.0)	31 (3.2)	25 (2.6)	30 (3.5)	55 (3.0)	68 (3.6)	0.13	<0.0001
Normal weight	844 (73.1)	445 (74.2)	399 (72.0)			734 (78.7)	765 (78.1)	784 (81.7)	704 (81.9)	1,382 (75.3)	1,605 (84.6)		
Overweight	211 (18.3)	101 (16.8)	110 (19.9)			132 (14.2)	160 (16.3)	134 (14.0)	101 (11.7)	339 (18.5)	188 (9.9)		
Obese	80 (6.9)	44 (7.3)	36 (6.5)			30 (3.2)	24 (2.5)	17 (1.8)	25 (2.9)	60 (3.3)	36 (1.9)		
Total	1,154	600	554			933	980	960	860	1836	1897		
Physical activity*, *n* (%)													
Low	123 (10.7)	54 (9.0)	69 (12.6)	0.06	0.14	283 (28.6)	314 (31.3)	386 (39.2)	380 (43.1)	503 (26.8)	861 (43.4)	0.19	<0.0001
Intermediate	97 (8.5)	50 (8.3)	47 (8.6)			215 (21.8)	190 (18.9)	191 (19.4)	176 (20.0)	368 (19.6)	404 (20.4)		
High	928 (80.8)	496 (82.7)	432 (78.8)			490 (49.6)	499 (49.8)	407 (41.4)	325 (36.9)	1,005 (53.6)	719 (36.2)		
Total	1,148	600	548			988	1,003	984	881	1876	1984		

*n*, number of subjects; %, percentage; BMI, body mass index; PA, physical activity. *PA in both surveys was classified as: low: <3 days a week; intermediate: 3 days a week; high: ≥4 days a week.

Of the total sample of HBSC adolescents aged 11–17 years, 3.3% were underweight and 16.7% had excessive weight (14.1% overweight and 2.6% obese), with a significantly higher proportion of overweight and obese adolescents among males (*p* < 0.001).

A significant association between PA levels and sex was also observed, with a higher proportion of poorly active adolescents among females (*p* < 0.001).

As described in the Methods, these findings refer to two distinct populations assessed with different tools and in different years, and should therefore be interpreted independently.

### 3.1 School health promotion practices and student behaviours

#### 3.1.1 OKkio alla Salute survey

Tables 3, 4 show the differences in selected children’s eating, PA and lifestyle habits between schools participating to the OKkio alla Salute survey, where nutrition and PA education and/or health promotion initiatives were implemented, and those where these interventions were not carried out.

**Table 3:** Comparison of children’s nutritional status and eating habits according to the presence of school-based nutrition education initiatives, parent-involving initiatives, and collaboration with the LHU.

Nutritional status and eating habits	School nutrition education				School initiative to promote healthy eating habits with parents				School initiative to promote healthy eating habits with LHU			
	No (*n* = 183)	Yes (*n* = 1,175)	Effect size	*p*-value	No (*n* = 1,011)	Yes (*n* = 281)	Effect size	*p*-value	No (*n* = 1,236)	Yes (*n* = 140)	Effect size	*p*-value
Weight status, *n* (%)												
Underweight	2 (1.3)	16 (1.6)	0.04	0.56*	10 (1.2)	6 (2.5)	0.05	0.37	18 (1.7)	1 (0.9)	0.03	0.89*
Normal weight	116 (76.3)	716 (72.7)			620 (73.4)	169 (69.3)			755 (72.7)	89 (76.7)		
Overweight	22 (14.5)	187 (19.0)			155 (18.3)	50 (20.5)			192 (18.5)	19 (16.4)		
Obese	12 (7.9)	66 (6.7)			60 (7.1)	19 (7.8)			73 (7.0)	7 (6.0)		
Breakfast, *n* (%)												
No	11 (7.3)	90 (9.0)	0.02	0.48	78 (9.2)	20 (8.0)	0.02	0.58	87 (8.3)	15 (12.8)	0.05	0.10
Yes	140 (92.7)	907 (91.0)			772 (90.8)	229 (92.0)			961 (91.7)	102 (87.2)		
Adequate breakfast, *n* (%)												
No breakfast	11 (7.3)	90 (9.1)	0.02	0.76	78 (9.2)	20 (8.1)	0.03	0.69	87 (8.3)	15 (12.8)	0.05	0.27
Yes, inadequate	54 (35.8)	342 (34.4)			285 (33.7)	90 (36.3)			362 (34.7)	38 (32.5)		
Yes, adequate	86 (56.9)	561 (56.5)			484 (57.1)	138 (55.7)			595 (57.0)	64 (54.7)		
Mid-morning snack, *n* (%)												
No	1 (0.7)	9 (0.9)	0.05	0.17*	7 (0.8)	3 (1.2)	0.23	<0.0001*	10 (1.0)	0 (0.0)	0.04	0.50*
Yes, inadequate	109 (73.6)	655 (66.2)			620 (73.6)	117 (47.6)			691 (66.6)	84 (71.8)		
Yes, adequate	38 (25.7)	325 (32.9)			216 (25.6)	126 (51.2)			336 (32.4)	33 (28.2)		
Fruits and/or vegetables consumption (≥ 5 times/day), *n* (%)												
No	152 (98.7)	1,011 (98.4)	0.01	1.00*	867 (98.4)	244 (98.4)	0.001	1.00*	1,063 (98.6)	117 (97.5)	0.03	0.41*
Yes	2 (1.3)	16 (1.6)			14 (1.6)	4 (1.6)			15 (1.4)	3 (2.5)		
Sugary and/or carbonated beverages (≥ once/day), *n* (%)												
No	116 (75.3)	773 (76.2)	0.01	0.82	658 (75.6)	187 (76.3)	0.01	0.82	800 (75.2)	100 (83.3)	0.06	0.05
Yes	38 (24.7)	242 (23.8)			212 (24.4)	58 (23.7)			264 (24.8)	20 (16.7)		

OKkio alla Salute survey, FVG region, 2023. *Fisher exact test. *n*, number of subjects; %, percentage; LHU, local health nits.

**Table 4:** Comparison of physical activity levels and sedentary behaviours among children according to the presence of school PA education initiatives, parent-involving initiatives and collaboration with the LHU.

Nutritional status, PA and sedentary habits	School physical activity education				School initiative to promote PA with parents				School initiative to PA with LHU			
	No (*n* = 32)	Yes (*n* = 1,344)	Effect size	*p*-value	No (*n* = 1,048)	Yes (*n* = 174)	Effect size	*p*-value	No (*n* = 1,353)	Yes (*n* = 23)	Effect size	*p*-value
Weight status, *n* (%)												
Underweight	0 (0.0)	19 (1.7)	0.03	0.76*	12 (1.4)	5 (3.5)	0.07	0.18	18 (1.6)	1 (5.0)	0.06	0.20*
Normal weight	21 (70.0)	823 (73.2)			635 (72.2)	108 (74.5)			832 (73.4)	12 (60.0)		
Overweight	6 (20.0)	205 (18.2)			165 (18.8)	25 (17.2)			205 (18.1)	6 (30.0)		
Obese	3 (10.0)	77 (6.9)			68 (7.7)	7 (4.8)			79 (7.0)	1 (5.0)		
Active children, *n* (%)												
No	2 (6.7)	127 (10.6)	0.02	0.76*	100 (10.6)	17 (11.0)	0.004	0.90	125 (10.3)	4 (20.0)	0.04	0.15*
Yes	28 (93.3)	1,076 (89.4)			841 (89.4)	138 (89.0)			1,088 (89.7)	16 (80.0)		
Outdoor play on day prior to survey, *n* (%)												
No	3 (10.0)	325 (28.9)	0.07	0.02*	248 (28.2)	51 (34.9)	0.05	0.10	316 (27.8)	12 (60.0)	0.09	0.002
Yes	27 (90.0)	800 (71.1)			633 (71.9)	95 (65.1)			819 (72.2)	8 (40.0)		
Sports on day prior to survey, *n* (%)												
No	18 (60.0)	559 (50.0)	0.03	0.28	444 (50.8)	82 (55.8)	0.04	0.26	568 (50.3)	9 (45.0)	0.01	0.64
Yes	12 (40.0)	560 (50.0)			430 (49.2)	65 (44.2)			561 (49.7)	11 (55.0)		
Walking or cycling to school, *n* (%)												
No	24 (80.0)	833 (73.4)	0.02	0.42	638 (71.9)	112 (75.2)	0.03	0.41	839 (73.3)	18 (90.0)	0.05	0.12*
Yes	6 (20.0)	302 (26.6)			249 (28.1)	37 (24.8)			306 (26.7)	2 (10.0)		
Screen time (>2 h/day), *n* (%)												
No	16 (55.2)	734 (66.4)	0.04	0.21	567 (66.2)	99 (68.3)	0.02	0.62	737 (66.0)	13 (72.2)	0.02	0.58
Yes	13 (44.8)	371 (33.6)			290 (33.8)	46 (31.7)			379 (34.0)	5 (27.8)		
TV in the child’s room, *n* (%)												
No	25 (78.1)	939 (79.6)	0.01	0.83	729 (79.5)	124 (80.0)	0.004	0.89	948 (79.6)	16 (80.0)	0.001	1.00*
Yes	7 (21.9)	240 (20.4)			188 (20.5)	31 (20.0)			243 (20.4)	4 (20.0)		

OKkio alla Salute survey, FVG region, 2023. *Fisher exact test. *n*, number of subjects; %, percentage; PA, physical activity; LHU, local health units.

In the 86.5% of schools that included nutrition education in the curricular activity (Table 3), the professional figure most frequently involved was the class teacher. Much less common was the involvement of other teachers (12%) or of health professionals (1%). Regarding PA, 97% of the schools implemented PA education (Table 4).

Initiatives to promote healthy eating habits and PA involved families in 21.7% and 14.2% of the sampled schools, respectively. Although LHUs were also contributing partners, they were only marginally involved in the implementation of nutrition education programs (10.2% of the schools) and in the promotion of PA (1.7% of the schools).

Overall, the majority (91.5%) of children had breakfast on the day of the interview, and for more than half of the children (56.8%) it was adequate. Breakfast consumption and quality were similar between schools that implemented any, or all, of the three nutrition-related activities (nutrition education, promotion of healthy eating habits involving either parents or LHUs) and those that implemented none, with no significant group differences.

Only 29% of the children consumed an adequate mid-morning snack. This behaviour was more frequent in schools reporting family involvement in healthy eating initiatives (*p* < 0.001). Moreover, only 1.5% of the children consumed fruits and/or vegetables ≥5 times a day, and 24.1% drank sugary and/or carbonated beverages every day, with no statistically significant differences between groups.

With regards to PA, 11% of the children reported no PA the previous day, 80.7% played outdoors and 50.8% participated in sports, on the day prior to the survey. As an additional measure of daily PA, data was also collected on routine walking or cycling to school. Active commuting was reported by 26.7% of children.

Furthermore, one out of three children exceeded the recommendation of maximum 2 hours of sedentary recreational screen time per day.

#### 3.1.2 HBSC survey

Table 5 shows adolescent outcomes by presence of written or unwritten school policies or guidelines on nutrition, PA and sedentary behaviour.

**Table 5:** Comparison of adolescents’ nutritional status, eating habits, PA levels and sedentary behaviours according to the presence of school nutrition-focused and physical activity-focused policies and guidelines.

Nutritional status, eating habits, PA and sedentary behaviours	School implementing nutrition-focused policies and guidelines				School implementing PA-focused policies and guidelines			
	No (*n* = 186)	Yes (*n* = 2,417)	Effect size	*p*-value	No (*n* = 70)	Yes (*n* = 2,533)	Effect size	*p*-value
Weight status, *n* (%)								
Underweight	5 (2.8)	70 (3.0)	0.03	0.57	2 (2.9)	73 (3.0)	0.02	0.78*
Normal weight	137 (77.0)	1851 (79.8)			54 (79.4)	1934 (79.6)		
Overweight	28 (15.7)	333 (14.4)			9 (13.2)	354 (14.5)		
Obese	8 (4.5)	65 (2.8)			3 (4.4)	70 (2.9)		
Breakfast, *n* (%)								
No	65 (35.1)	942 (39.0)	0.02	0.30	—	—		—
Yes	120 (64.9)	1,473 (61.0)			—	—		
Fruit (≥ once/day), *n* (%)								
No	146 (78.5)	1,636 (67.7)	0.06	0.002	—	—		—
Yes	40 (21.5)	779 (32.3)			—	—		
Vegetable (≥ once/day), *n* (%)								
No	128 (68.8)	1,482 (61.4)	0.04	0.05	—	—		—
Yes	58 (31.2)	931 (38.6)			—	—		
Sugary and/or carbonated beverages (≥ once/day), *n* (%)								
No	166 (89.2)	2,196 (91.0)	0.02	0.42	—	—		—
Yes	20 (10.8)	217 (9.0)			—	—		
Moderate-intense physical activity (≥4 days a week), *n* (%)								
No	—	—		—	46 (65.7)	1,382 (54.9)	0.04	0.07
Yes	—	—			24 (34.3)	1,134 (45.1)		
Vigorous physical activity (≥3 days a week), *n* (%)								
No	—	—		—	38 (55.1)	1,171 (46.5)	0.03	0.16
Yes	—	—			31 (44.9)	1,345 (53.5)		
Time spent playing video games/PC/tablet/mobile phone (>2 h/day), *n* (%)								
No	—	—		—	50 (71.4)	1867 (73.9)	0.01	0.65
Yes	—	—			20 (28.6)	660 (26.1)		
Time spent watching TV (>2 h/day), *n* (%)								
No	—	—		—	49 (71.0)	1939 (76.8)	0.02	0.26
Yes	—	—			20 (29.0)	586 (23.2)		

*HBSC, FVG region, 2022. Fisher exact test. *n*, number of subjects; %, percentage; PA, physical activity.

For nutrition, 21.6% of school principals reported that their schools implemented written policies or guidelines, while in 71.6% of schools’ policies or guidelines were present but unwritten. Only 6.8% of schools reported having neither written nor unwritten policies to promote healthy eating.

For PA, 37.8% of schools reported having written policies or guidelines, 58.1% unwritten, and 4.1% none.

A total of 74.3% of school principals reported that students were “almost always” or “often” involved in organizing and developing school health promotion initiatives.

Overall, 38.3% of adolescents did not eat breakfast every day and 9.2% drunk sugary and/or carbonated beverages daily, these behaviours did not differ substantially whether or not the school had nutrition related polies in place.

The majority of adolescents ate fruit (67.6%) and vegetables (61.2%) less than once a day. A single statistically significant difference was identified: fruit consumption was more frequent among adolescents attending schools with nutrition-related policies or guidelines (*p* = 0.002).

Regarding PA, 55.3% of the adolescents who participated in the HBSC surveillance practiced moderate-intense PA less than 3 days a week and 46% practiced vigorous PA 2 days a week or less. Furthermore, slightly more than half met the recommendations of not exceeding 2 hours a day of screen time. Specifically, 56.8% spent less than 2 hours per day playing video games or using a PC, tablet, or mobile phone, and 55.4% spent less than 2 hours per day watching TV, DVDs, or online videos. No significant differences were found in PA or sedentary behaviours between students attending schools with or without related policies or guidelines.

In both surveys, weight status distribution was similar across schools, regardless of whether or not they reported having health promotion practices or policies, and no statistically significant associations were observed.

## 4 Discussion

This study provides data on the prevalence of weight status, on dietary habits and PA and sedentary behaviours among students aged 8 to 17 in primary and secondary schools in the FVG region, and describes how these outcomes was associated to the presence or absence of school-reported health promotion practices and policies.

### 4.1 Nutritional status

The results indicate that overweight and obesity remain prevalent among school-aged children and adolescents, particularly among boys, thereby confirming previous national and international findings. Our prevalence data are in line with those from the other northern Italian regions and show a substantially lower prevalence of excessive weight compared to southern Italy, confirming the national north–south gradient (2, 27–29).

Adolescence represents a critical developmental stage that marks the transition from childhood to adulthood and is characterized by profound psychological, biological, and hormonal changes, as well as significant shifts in social roles (30, 31). In line with prior reports, our findings indicate a decline in the prevalence of overweight and obesity from pre-pubertal to pubertal stages (8, 27, 32). Although self-reported anthropometric measures among adolescents may be underestimated as a result of reporting and social desirability biases (33–35), our results are consistent with national (36) and international studies (37).

The decrease in overweight and obesity prevalence may be due to hormonal changes that occur as boys and girls approach puberty (38). In addition, teenagers tend to pay more attention to their physical appearance and to put more effort into controlling their weight. Increased stress and pressure associated with middle and high school may also contribute to weight loss in this age group (39). In terms of differences in BMI categories between males and females, the higher prevalence of overweight and obesity in males we observed is consistent with previous literature (40).

### 4.2 Dietary habits and physical activity

While among younger children daily breakfast consumption was high and in line with comparable Italian (22) and international contexts (41), it sharply declined in adolescence, confirming a concerning trend that is already well documented in the literature (3, 29). Daily consumption of fruits and vegetables remains critical in children and low among adolescents. This is in line with the WHO-HBSC 2022 report (3), and with findings across Europe that highlight widespread challenges in meeting the recommended dietary guidelines (42).

PA levels among younger children are high, yet only about half regularly participate in sports. Active commuting to school is limited, the main barriers reported by parents being excessive distance to school, lack of parent’s time and road safety issues. The cross-sectional nature of our study does not allow us to assess the role of existing school initiatives in influencing the behavioural patterns described above. However, our findings can point to areas where school-based strategies could be further developed to support daily PA but also highlight missed opportunities, such as initiatives to promote walking and cycling to school with well-documented benefits for both health and sustainability (11). Among adolescents, over half fail to meet recommended levels of moderate-intense and vigorous PA, which is consistent with European surveillance data. Disparities between sexes also align with global trends, with higher inactivity among females (43, 44). These differences underscore the relevance of gender-sensitive approaches identified in previous literature (45, 46), although the present study does not address the potential benefits of such approaches.

### 4.3 School environment

Our results underscore the role of schools health promotion: with most of the FVG primary schools integrating nutrition and PA education in their curricula. However, these activities are largely delivered by classroom teachers, and the involvement of external professionals or stakeholders, particularly LHUs, is lower than the average of the other regions of Northern Italy (12% vs. 16.7%) and of the national data (23.5%) (2). This approach reflects the Regional strategy that prioritizes teacher-led implementation to ensure equity and universal access, as reported in the Regional guidance documents (47). The limited contribution of external stakeholders should therefore be seen as an executive choice rather than an organizational shortcoming.

In our sample, parental engagement in school initiatives is limited, but where present, it is associated with a higher proportion of primary school children consuming an adequate mid-morning snack (*p* < 0.001). While this association cannot be interpreted as causal evidence that parental involvement directly drives behavioural change, it aligns with the existing literature that suggests that active parental involvement can enhance the effectiveness of school-based interventions targeting children’s dietary behaviours (48, 49).

A nuanced picture emerges from the HBSC data regarding school policies. Only a minority of schools report having written policies for promoting healthy eating and PA, while a larger proportion rely on unwritten guidelines. This reliance on non-formalized approaches may be associated with variability in the consistency, accountability, and reach of school-based practices. Previous studies suggest that structured, written policies are more likely to be implemented into the school setting (7).

Student involvement in shaping health promotion initiatives is relatively high in FVG. This is a positive finding, as youth engagement is recognized as a key determinant in the success of school-based health promotion programs (50). However, further investigations are needed to understand whether students are engaged through meaningful participation or more symbolic involvement.

Across both surveys, most student behaviours and weight-related outcomes did not differ significantly according to the presence or absence of reported school practices or policies, except for an increased prevalence of adequate mid-morning snacks in children when parents are involved in healthy eating school-based initiatives. However, the absence of statistically significant associations in our data does not imply that school health promotion strategies are ineffective; rather, it likely reflects the heterogeneity of practices and policies across schools, the use of binary exposure classifications, the lack of information on implementation fidelity or duration, potential residual confounding, and the limited sensitivity of cross-sectional surveillance data to detect small effects in the absence of temporal sequencing.

Based on the literature, interventions that yield significant impact, typically include a combination of actions aimed at increasing PA, decreasing sedentary behaviours, and improving dietary habits (51) and involve parents through workshops, take-home activities, and regular communication (52). The duration of interventions also appears to play a relevant role. There is, in fact, evidence supporting interventions lasting at least an entire school year that include multi-sectoral collaboration, parental engagement and clear policy frameworks (14, 53–55).

### 4.4 Strength and limitations

A major strength of this study is the large and representative sample of both primary and secondary school students, which allows for robust comparisons and generalizability within the region. Moreover, the study provides descriptive evidence on the presence of specific school interventions and their relationship with health-related behaviours, although these findings cannot be interpreted in causal terms.

Our study needs to be interpreted in the light of some limitations. First, the cross-sectional design of the study precludes inferences regarding temporal ordering or causality. Second, the structure of the questionnaires did not allow to obtain detailed information on the types of programs and their contents, and the variability in intervention intensity and implementation quality across schools was not systematically measured. Consequently, exposure measurement was necessarily coarse and potentially affected by misclassification, which may have contributed to few statistically significant associations, such as the low prevalence of children that consume fruits and/or vegetables ≥5 times a day.

In addition, the use of self-reported data, particularly among adolescents, introduces potential reporting biases, especially for sensitive variables like body weight, PA and sedentary behaviours. In addition, since the two surveys were designed to target different age groups, some questions were worded differently, and this may have further accentuated the differences observed between the two populations. Lastly, while it is likely that the relatively low level of collaboration with parents and LHUs reported by most schools may have limited the scope of the school-based activities, the observational design of our study does not allow to determine the extent its influence on the behaviours observed.

## 5 Conclusion

In conclusion, the results of our cross-sectional regional analysis indicate that, although school-based health promotion practices are widely implemented in the FVG region, their observable associations with children’s and adolescents’ weight status and health behaviours are limited. The present study provides a descriptive insight into health promotion in the school setting in FVG, providing a framework to support the development of tailored evaluation tools capable of assessing implementation processes and potential impacts on health behaviours. Enhanced multi-sectoral collaboration, stronger parental involvement, and a more rigorous policy enforcement are necessary to achieve meaningful improvements in childhood obesity prevention. Addressing these factors will be crucial to supporting the WHO’s recommendations and advancing toward healthier school environments across Europe.

## Data Availability

The datasets presented in this article are not readily available because the data analysed in this study is subject to the following licenses/restrictions: The data used in this study are drawn from the Italian OKkio alla Salute and HBSC surveillance systems, which is managed by the Istituto Superiore di Sanità. Due to confidentiality agreements and institutional regulations, the raw data cannot be shared publicly. Requests for access can be directed to the corresponding author, who will liaise with the data custodians as appropriate. Requests to access the datasets should be directed to federica.concina@burlo.trieste.it.

## Group member of HBSC FVG group

Luana Sandrin (Central Directorate for Health, Social Policies, and Disability of the FVG Regional Authority); Livio Consonni (Regional Education Authority); ASUGI: Roberta Fedele (Rappresentative), Emanuela Occoni, Alessandra Pahor; ASUGI-Isontino Area: Claudia Loi (Rappresentative); ASUFC Donatella Belotti (Rappresentative), Martina Piera Lupo, Danila Dosa; ASFO: Anna Clara Guastaferro (Rappresentative), Carmen Zampis.

## Group member of OKkio alla Salute FVG group

Luana Sandrin (Central Directorate for Health, Social Policies, and Disability of the FVG Regional Authority); Antonio Screti (Regional Education Authority); ASUGI: Tiziana Longo (Rappresentative), Emanuela Occoni, Daniela Steinbock, Marilena Geretto, Alessandra Pahor; ASUGI-Isontino Area: Jetlira Begaj (Rappresentative), Rosa Padovano, Tamara Omasta; ASUFC: Danila Dosa (Rappresentative), Andrea Anastasia Ballaminut, Martina Piera Lupo, Manuela Mauro, Emanuela Milio; ASFO: Stefania Pilan (Rappresentative), Lucrezia Antoniolli, Matteo Bellu, Michela Beltrame, Francesca Berri, Daniela Bertolo, Eddy Galiazzo, Jessica Giordari, Jessica Greguol, Anna Clara Guastaferro, Silvia Moras, Michela Muzzin, Raffaella Pittana, Sandra Polese, Paola Roà, Jessica Sorentini, Daniela Sut, Maria Teresa Todesco, Alessandra Volpicina, Carmen Zampis.

## References

[1] World Health Organization - European Region. WHO European childhood obesity surveillance initiative (COSI) brief review of results from round 6 of 2022–2024. Copenhagen: World Health Organization (2024).

[2] Nardone P Ciardullo S Spinelli A Mandolini D Antonio Salvatore M Andreozzi S. Stato ponderale e stili di vita di bambine e bambini: i risultati di OKkio alla SALUTE 2023. Rome: Istituto Superiore di Sanità (2025).

[3] Rakić JG Hamrik Z Dzielska A Felder-Puig R Oja L Bakalár P . A focus on adolescent physical activity, eating behaviours, weight status and body image in Europe, Central Asia and Canada: health behaviour in school-aged children international report from the 2021/2022 survey. Copenhagen: World Health Organization (2024).

[4] World Health Organization. Report of the commission on ending childhood obesity. Geneva: World Health Organization (2016).

[5] Arai L Panca M Morris S Curtis-Tyler K Lucas PJ Roberts HM. Time, monetary and other costs of participation in family-based child weight management interventions: qualitative and systematic review evidence. PLoS One. (2015) 10:e0123782. doi: 10.1371/journal.pone.0123782, 25853729 PMC4390145

[6] Alhelal A AlSalem MS Alasmari FMA Alqarni SA Alamri RMA Alshahrani RAA . Effectiveness of school-based interventions for preventing obesity in children: a narrative review. Cureus. (2024) 16:e75104. doi: 10.7759/cureus.75104 39759640 PMC11698265

[7] Story M Nanney MS Schwartz MB. Schools and obesity prevention: creating school environments and policies to promote healthy eating and physical activity In: The Milbank quarterly. Hoboken, NJ: Wiley Periodicals Inc (2009) 10.1111/j.1468-0009.2009.00548.x PMC2879179 19298416

[8] World Health Organization. Report of the commission on ending childhood obesity. Implementation plan: executive summary. 2017. Available online at: http://apps.who.int/iris (Accessed June 23, 2025)

[9] Pulimeno M Piscitelli P Colazzo S Colao A Miani A. School as ideal setting to promote health and wellbeing among young people. Health Promot Perspect. (2020) 10:316–34. doi: 10.34172/hpp.2020.50 33312927 PMC7723000

[10] Masini A Salussolia A Anastasia A Grao-Cruces A Soldà G Zanutto G . Evaluation of school-based interventions including homework to promote healthy lifestyles: a systematic review with meta-analysis. J Public Health. (2024) 34:23–39. doi: 10.1007/s10389-024-02239-6

[11] Larouche R Saunders TJ Faulkner GEJ Colley R Tremblay M. Associations between active school transport and physical activity, body composition, and cardiovascular fitness: a systematic review of 68 studies. J Phys Act Health. (2014) 11:206–27. doi: 10.1123/jpah.2011-0345, 23250273

[12] Homs C Berruezo P Según G Estrada L de Bont J Riera-Romaní J . Family-based intervention to prevent childhood obesity among school-age children of low socioeconomic status: study protocol of the FIVALIN project. BMC Pediatr. (2021) 21:246. doi: 10.1186/s12887-021-02697-x 34020614 PMC8139065

[13] Bean MK Caccavale LJ Adams EL Burnette CB Larose JG Raynor HA . Parent involvement in adolescent obesity treatment: a systematic review. Pediatrics. (2020) 146:20193315. doi: 10.1542/peds.2019-3315 PMC7461263 32839242

[14] Butscher F Ellinger J Singer M Mall C. Influencing factors for the implementation of school-based interventions promoting obesity prevention behaviors in children with low socioeconomic status: a systematic review. Implement Sci Commun. (2024) 5:12. doi: 10.1186/s43058-024-00548-1, 38347649 PMC10860312

[15] Bennett S Woods T Liyanage WM Smith Duane L. A simplified general method for cluster-sample surveys of health in developing countries. World Health Stat Q. (1991) 44:98–106. 1949887

[16] Binkin N Fontana G Lamberti A Cattaneo C Baglio G Perra A . A national survey of the prevalence of childhood overweight and obesity in Italy. Obes Rev. (2010) 11:2–10. doi: 10.1111/j.1467-789X.2009.00650.x 19761508

[17] Spinelli A Lamberti A Baglio G Andreozzi S Galeone D. OKkio alla SALUTE: sistema di sorveglianza su alimentazione e attività fisica nei bambini della scuola primaria. Risultati 2008. Roma: Istituto Superiore di Sanità EpiCentro - L'epidemiologia per la sanità pubblica (2009).

[18] Inchley J CDCA& SO. Health behaviour in school-aged children (HBSC) study protocol: background, methodology and mandatory items for the 2017/18 survey. St Andrews: CDCA& SO (2018).

[19] Cole TJ Flegal KM Nicholls D Jackson AA. Body mass index cut offs to define thinness in children and adolescents: international survey. BMJ. (2007) 335:194–7. doi: 10.1136/bmj.39238.399444.55, 17591624 PMC1934447

[20] Cole TJ Bellizzi MC Flegal KM Dietz WH. Establishing a standard definition for child overweight and obesity worldwide: international survey. BMJ. (2000) 320:1240–3. doi: 10.1136/bmj.320.7244.1240 10797032 PMC27365

[21] CREA - Centro di ricerca Alimenti e nutrizione. Linee Guida per una Sana Alimentazione - Revisione 2018. Rome: CREA - Centro di ricerca Alimenti e nutrizione (2019).

[22] Ciardullo S Salvatore MA Mandolini D Spinelli A Bucciarelli M Andreozzi S . Trend in breakfast consumption among primary school children in Italy. Nutrients. (2023) 15:4632. doi: 10.3390/nu15214632, 37960286 PMC10647676

[23] World Health Organization 2020 WHO guidelines on physical activity and sedentary behaviour. Available online at: http://apps.who.int/iris (Accessed June 23, 2025)

[24] Gentile DA Berch ON Choo H Khoo A Walsh DA. Bedroom media: one risk factor for development. Dev Psychol. (2017) 53:2340–55. doi: 10.1037/dev0000399, 28945440

[25] Musić Milanović S Buoncristiano M Križan H Rathmes G Williams J Hyska J . Socioeconomic disparities in physical activity, sedentary behavior and sleep patterns among 6- to 9-year-old children from 24 countries in the WHO European region. Obes Rev. (2021) 22. doi: 10.1111/obr.13209 34235843

[26] Torsheim T Cavallo F Levin KA Schnohr C Mazur J Niclasen B . Psychometric validation of the revised family affluence scale: a latent variable approach. Child Indic Res. (2016) 9:771–84. doi: 10.1007/s12187-015-9339-x 27489572 PMC4958120

[27] Stival C Lugo A Barone L Fattore G Odone A Salvatore S . Prevalence and correlates of overweight, obesity and physical activity in Italian children and adolescents from Lombardy, Italy. Nutrients. (2022) 14:2258. doi: 10.3390/nu14112258 35684058 PMC9182936

[28] Wijnhoven TM Van Raaij JM Spinelli A Starc G Hassapidou M Spiroski I . WHO European childhood obesity surveillance initiative: body mass index and level of overweight among 6–9-year-old children from school year 2007/2008 to school year 2009/2010. BMC Public Health. (2014) 14:806. doi: 10.1186/1471-2458-14-806 25099430 PMC4289284

[29] Lazzeri G Simi R Ciardullo S Pierannunzio D Bacigalupo I Andreozzi S La Sorveglianza HBSC-Italia 2022 Health Behaviour in School-aged Children: le abitudini alimentari, lo stato ponderale e l’attività fisica degli adolescenti. 2024. Available online at: https://www.iss.it/documents/20126/6703853/the+HBSC-Italia+2022+-+Health+Behaviour+in+School-aged+Children+Surveillance+eating+habits%2C+weight+status+and+physical+activity+of+adolescents.pdf/c5440e30-1abf-fe4e-ea4e-dedb831af6a3?t=1724833825539 (Accessed June 23, 2025)

[30] Sawyer SM Afi RA Bearinger LH Blakemore SJ Dick B Ezeh AC . Adolescence: a foundation for future health. Lancet. (2012) 379:1630–70. doi: 10.1016/S0140-6736(12)60072-5 22538178

[31] Patton GC Sawyer SM Santelli JS Ross DA Afifi R Allen NB . Our future: a lancet commission on adolescent health and wellbeing. Lancet. (2016) 387:2423–78. doi: 10.1016/S0140-6736(16)00579-1, 27174304 PMC5832967

[32] Lobstein T Baur L Uauy R. Obesity in children and young people: a crisis in public health. Obes Rev. (2004) 5:4–104. doi: 10.1111/j.1467-789X.2004.00133.x 15096099

[33] Bostrom G Diderichsen F. Socioeconomic differentials in misclassification of height, weight and body mass index based on questionnaire data. Int J Epidemiol. (1997) 26:860–6. doi: 10.1093/ije/26.4.860 9279620

[34] Lin CJ Deroo LA Jacobs SR Sandler DP. Accuracy and reliability of self-reported weight and height in the sister study. Public Health Nutr. (2012) 15:989–99. doi: 10.1017/S1368980011003193, 22152926 PMC3511620

[35] Galfo M Censi L D’Addezio L Martone D Roccaldo R. Validity of self-reported weight, height and BMI in Italian adolescents for assessing prevalence of overweight/obesity. Clin Nutr Metab. (2018) 1:1–7. doi: 10.15761/CNM.1000101

[36] Istituto Nazionale di Statistica. Stili di vita dei bambini e dei ragazzi. Anni 2017–2018. 2019. Available online at: https://www.istat.it/it/archivio/234930 (Accessed June 23, 2025)

[37] Ng M Gakidou E Lo J Abate YH Abbafati C Abbas N . Global, regional, and national prevalence of adult overweight and obesity, 1990–2021, with forecasts to 2050: a forecasting study for the global burden of disease study 2021. Lancet. (2025) 405:813–38. doi: 10.1016/S0140-6736(25)00355-1 40049186 PMC11920007

[38] Veldhuis JD Roemmich JN Richmond EJ Rogol AD Lovejoy JC Sheffield-Moore M . Endocrine control of body composition in infancy, childhood, and puberty. Endocr Rev. (2005) 26:114–46. doi: 10.1210/er.2003-0038 15689575

[39] Zhang X Liu J Ni Y Yi C Fang Y Ning Q . Global prevalence of overweight and obesity in children and adolescents: a systematic review and meta-analysis. JAMA Pediatr. (2024) 178:800–13. doi: 10.1001/jamapediatrics.2024.1576 38856986 PMC11165417

[40] Shah B Tombeau Cost K Fuller A Birken CS Anderson LN. Sex and gender differences in childhood obesity: contributing to the research agenda. BMJ Nutr Prev Health. (2020) 3:387–90. doi: 10.1136/bmjnph-2020-000074, 33521549 PMC7841817

[41] Rampersaud GC Pereira MA Girard BL Adams J Metzl JD. Breakfast habits, nutritional status, body weight, and academic performance in children and adolescents. J Am Diet Assoc. (2005) 105:743–60. doi: 10.1016/j.jada.2005.02.007 15883552

[42] World Health Organization. Healthy diet—2020 2020. Available online at: https://www.who.int/news-room/fact-sheets/detail/healthy-diet (Accessed June 24, 2025)

[43] Guthold R Stevens GA Riley LM Bull FC. Global trends in insufficient physical activity among adolescents: a pooled analysis of 298 population-based surveys with 1.6 million participants. Lancet Child Adolesc Health. (2020) 4:23–35. doi: 10.1016/S2352-4642(19)30323-2, 31761562 PMC6919336

[44] Kretschmer L Salali GD Andersen LB Hallal PC Northstone K Sardinha LB . Gender differences in the distribution of children’s physical activity: evidence from nine countries. Int J Behav Nutr Phys Act. (2023) 20:103. doi: 10.1186/s12966-023-01496-0, 37667391 PMC10478357

[45] Schlund A Reimers AK Bucksch J Linder S Demetriou Y. Sex/gender considerations in school-based interventions to promote children’s and adolescents’ physical activity: a systematic review. Ger J Exerc Sport Res. (2021) 51:257–68. doi: 10.1007/s12662-021-00724-8

[46] McKenzie TL. Physical activity within school contexts: the bigger bang theory. Kinesiol Rev. (2019) 8:48–53. doi: 10.1123/kr.2018-0057

[47] Friuli Venezia Giulia Region. Piano-regionale-della-prevenzione-2021–2025. Available online at: https://invecchiamentoattivo.regione.fvg.it/export/sites/invecchiamento-attivo/it/allegati/Piano-regionale-della-prevenzione-2021-2025.pd. (Accessed December 19, 2025)

[48] Golan M Crow S. Parents are key players in the prevention and treatment of weight-related problems. Nutr Rev. (2004) 62:39–50. doi: 10.1111/j.1753-4887.2004.tb00005.x 14995056

[49] Knai C Petticrew M Mays N Capewell S Cassidy R Cummins S . Systems thinking as a framework for analyzing commercial determinants of health. Milbank Q. (2018) 96:472–98. doi: 10.1111/1468-0009.12339 30277610 PMC6131339

[50] Langford R Bonell CP Jones HE Pouliou T Murphy SM Waters E . The WHO health promoting school framework for improving the health and well-being of students and their academic achievement. Cochrane Database Syst Rev. (2014) 2014:CD008958. doi: 10.1002/14651858.CD008958.pub2 24737131 PMC11214127

[51] Nally S Carlin A Blackburn NE Baird JS Salmon J Murphy MH . The effectiveness of school-based interventions on obesity-related behaviours in primary school children: a systematic review and meta-analysis of randomised controlled trials. Children. (2021) 8:489. doi: 10.3390/children8060489, 34201145 PMC8228296

[52] Van De Kolk I Verjans-Janssen SRB Gubbels JS Kremers SPJ Gerards SMPL. Systematic review of interventions in the childcare setting with direct parental involvement: effectiveness on child weight status and energy balance-related behaviours. Int J Behav Nutr Phys Act. (2019) 16:110. doi: 10.1186/s12966-019-0874-6, 31752917 PMC6873502

[53] Drouka A Brikou D Causeret C Al Ali Al Malla N Sibalo S Ávila C . Effectiveness of school-based interventions in Europe for promoting healthy lifestyle behaviors in children. Children. (2023) 10:1676. doi: 10.3390/children10101676, 37892339 PMC10605522

[54] Zurc J Laaksonen C. Effectiveness of health promotion interventions in primary schools—a mixed methods literature review. Healthcare. (2023) 11:1817. doi: 10.3390/healthcare11131817, 37444651 PMC10340694

[55] Rozga M Handu D. Nutrition interventions for pediatric obesity prevention: an umbrella review of systematic reviews. Nutrients. (2023) 15:5097. doi: 10.3390/nu15245097, 38140356 PMC10745722

