# Supplementary material for: Assessing the association between health promotion initiatives and student nutritional status and lifestyle: a regional observational study from Italy
Source: Front Public Health. 2026 Feb 16;14:1750624. doi: 10.3389/fpubh.2026.1750624 (PMC12950776; doi:10.3389/fpubh.2026.1750624)
Supplement: Supplementary file 1 [file Supplementary_file_1.doc]

| **Table S1. Items and variables derived from OKkio alla Salute and HBSC questionnaires organised by survey source and respondent.** | |
| --- | --- |
| 1. **OKkio alla Salute –School principal’s questionnaire** | |
| Curricular nutrition education (more than one answer possible) | - No - Yes, with the class teacher - Yes, with designated teachers - Yes, with external teacher - Yes, with health care provider |
| The Ministry of Education, University and Research is promoting children's PA in elementary school. With respect to this, the school: (more than one answer possible) | - Has a structured and effective training curriculum - Implements teacher training on this curriculum - Provides or uses external experts - Provides for the assessment of children's motor skills - Has not yet undertaken improvement activities |
| Active involvement of parents in the planned or implemented activities to promote healthy eating habits | - Yes - No |
| Active involvement of parents in the planned or implemented activities to promote physical activity | - Yes - No |
| Collaboration with LHU's for the implementation of health promotion programs (more than one answer possible) | - Yes, nutrition education - Yes, promotion of PA - No |
| 1. **OKkio alla Salute –Parent’s questionnaire** | |
| Breakfast consumption | - Every day - 4-6 days a week - 1-3 days a week - Never |
| Fruit consumption | - 4 or more times a day - 2-3 times a day - Once a day - 4-6 days a week - 1-3 days a week - Less than once a week |
| Vegetable consumption | - 4 or more times a day - 2-3 times a day - Once a day - 4-6 days a week - 1-3 days a week - Less than once a week |
| Sugary and/or carbonated beverages | - Never - Less than once a week - 1-3 days a week - 4-6 days a week - Once a day - More than once a day |
| Sports for at least 1 hour a day, outside of school hours. | - Never - 1 day/week - 2 days/week - 3 days/week - 4 days/week - 5 days/week - 6 days/week - Every day |
| Outdoor play for at least 1 hour a day, outside of school hours. | - Never - 1 day/week - 2 days/week - 3 days/week - 4 days/week - 5 days/week - 6 days/week - Every day |
| Playing video games, PC/Tablet/mobile phone | - Number of hours ___and minutes __ per day on a normal school day - Number of hours ___and minutes __ per day at the weekend - Never |
| Watching TV | - Number of hours ___and minutes __ per day on a normal school day - Number of hours ___and minutes __ per day at the weekend - Never |
| TV in child’s room | - Yes - No |
| 1. **OKkio alla Salute –Children’s questionnaire** | |
| Breakfast on the day of the survey | - Yes - No |
| Food choices for breakfast (more than one answer possible) | - Milk - Milk with cocoa or barley - Tea - Juice - Freshly squeezed orange juice - Croissant - Sweet bread - Snack - Cookies - Sandwich or toast - Bread and jam - Bread and chocolate spread - Pizza or focaccia bread - Crackers or breadsticks - Rusks - Cake or pie - Yoghurt - Cereals - Fruit - Eggs - Other |
| Mid-morning snack on the day of the survey | - Yes - No |
| Food choices for mid-morning snack (more than one answer possible) | - Juice - Soft drink (cola or orange soda) - Tea - Snack or cereal bar - Yoghurt - Sweet snack or cake - Cookies - Sandwich or toast - Crackers or breadsticks - Rusks - Pizza or focaccia bread - Croissant - Sweet bread - Chips - Fruit |
| Outdoor play on the day prior to the survey | - Yes, at school - Yes, after school - No |
| Sports on the day prior to the survey | - Yes, at school - Yes, after school - No |
| Getting to school | - On foot - By bike/scooter/skates - By bus/school bus - By car/motorcycle |
| Returning from school | - On foot - By bike/scooter/skates - By bus/school bus - By car/motorcycle |
| Screen time before school | - Yes - No |
| Videogames or games on computer/tablet/cell-phone in the afternoon the day prior to the survey | - Yes - No |
| TV in the afternoon the day prior to the survey | - Yes - No |
| Videogames or games on computer/tablet/cell-phone after dinner the day prior to the survey | - Yes - No |
| TV after dinner the day prior to the survey | - Yes - No |
| 1. **HBSC –School principal’s questionnaire** | |
| School policies or guidelines on healthy eating habits | - No - Yes, formal policies/guidelines - Yes, informal policies/guidelines |
| School policies or guidelines on physical activity | - No - Yes, policies/guidelines formal - Yes, policies/guidelines in formal |

| Students invited/involved to develop and design health promotion activities/initiatives | - Almost always - Often - Sometimes - Rarely - Never |
| --- | --- |
| 1. **HBSC –Adolescents’ questionnaire** | |
| Breakfast consumption on week-days | - Never - 1 day - 2 days - 3 days - 4 days - 5 days |
| Breakfast consumption at the weekend | - Never - 1 day - 2 days |
| Fruit consumption | - Never - Less than once a week - Once a week - 2-4 days a week - 5-6 days a week - Once daily - More than once a day |
| Vegetable consumption | - Never - Less than once a week - Once a week - 2-4 days a week - 5-6 days a week - Once daily - More than once a day |
| Sugary and/or carbonated beverages consumption | - Never - Less than once a week - Once a week - 2-4 days a week - 5-6 days a week - Once daily - More than once a day |
| Moderate PA for at least 1 hour | - Never - 1 day/week - 2 days/week - 3 days/week - 4 days/week - 5 days/week - 6 days/week - Every day |
| Vigorous PA (substantial increase of heart and breathing rates) | - Every day - 4-6 times a week - 3 times a week - 2 times a week - Once a week - Once a month - Less once a month - Never |
| Playing video games, PC/Tablet/mobile phone | - Never - About half an hour/day - About 1 hour/day - About 2 hours/day - About 3 hours/day - About 4 hours/day - About 5 hours/day - About 6 hours/day - About 7 hours/day |
| Using the PC/Tablet/ mobile phone to be on social networks (e.g. Facebook, Twitter, Snapchat, etc.) | - Never - About half an hour/day - About 1 hour/day - About 2 hours/day - About 3 hours/day - About 4 hours/day - About 5 hours/day - About 6 hours/day - About 7 hours/day |
| Watching TV/DVDs or videos, including those on websites such as You Tube, etc. | - Never - About half an hour/day - About 1 hour/day - About 2 hours/day - About 3 hours/day - About 4 hours/day - About 5 hours/day - About 6 hours/day - About 7 hours/day |
